# DL-3-n-butylphthalide Increases Collateriogenesis and Functional Recovery after Focal Ischemic Stroke in Mice

**DOI:** 10.14336/AD.2020.1226

**Published:** 2021-10-01

**Authors:** Zheng Zachory Wei, Dongdong Chen, Matthew Joong H Lee, Yingying Zhao, Xiaohuan Gu, Shan Ping Yu, Ling Wei

**Affiliations:** Department of Anesthesiology, Emory University School of Medicine, Atlanta, GA, USA.

**Keywords:** ischemic stroke, DL-3-n-butylphthalide, arteriogenesis, collateral artery, regeneration, functional recovery

## Abstract

Recent evidence indicates that collateral circulation is critical for the outcome of ischemic stroke. DL-3-n-butylphthalide (NBP), a synthesized compound based on an extract from seeds of celery *Apium graveolens Linn*, has been used as a therapeutic drug, showing multiple neuroprotective and regenerative activities. A potential effect of NBP on collateral arterial regulation is unknown. We examined the effects of NBP on arteriogenesis of collateral arteries *in vitro* and a mouse ischemic stroke model. In cultures of mouse iPS cell-derived vascular progenitors, NBP (10 μM) significantly increased α-smooth muscle actin (αSMA)/CD-31 co-labeled cells and the expression of newly formed vasculature marker PDGFRα. A sensorimotor cortex ischemia was induced in transgenic mice expressing αSMA-GFP that allowed direct observation of arterial vasculatures in brain regions. NBP (80 mg/kg) was intranasally delivered 1 hr after stroke and once daily for 14 days. To label proliferating cells, 5-Bromo-2’-deoxyuridine (BrdU, 50 mg/kg, i.p.) was administrated every day from 3 days after stroke. Western blotting of peri-infarct tissue detected increased expressions of VEGF, Ang-1 and reduced nNOS level in NBP-treated mice. The NBP treatment significantly increased αSMA/BrdU co-labeled cells, the diameter of ipsilateral collaterals, and arterial area in ischemic and peri-infarct regions examined 14 days after stroke. Examined 3 days after stroke, NBP prevented functional deficits in the cylinder test and corner test. The NBP treatment of 14 days improved the local cerebral blood flow (LCBF) and functional performance in multiple tests. Thus, NBP promotes collateriogenesis, short and long-term structural and functional improvements after ischemic stroke.

Stroke is a leading cause of human death and disability including long-term functional deficits [1]. Clinical treatments to promote cell survival for ischemic stroke have been limited to thrombolytic therapy using tissue plasminogen activator (tPA) or thrombectomy by mechanical means, which can benefit only a limited number of patients [2-5]. On the other hand, regenerative treatments after stroke have emerged as a promising therapy for tissue repair and functional recovery [6, 7]. Most investigations have been focused on promoting peri-infarct angiogenesis, neurogenesis, and neuroplasticity [8, 9]. Several clinical trials of thrombectomy identified that the collateral circulation around the ischemic core region is an important factor in determining outcomes of stroke patients [4, 10, 11]. In acute ischemic stroke, collateral circulation acts as a mechanism of rescuing the local blood flow to the brain region that is ischemic or at risk of progression into ischemia. Therefore, promoting collateral blood flow and collateriogenesis became new and promising therapeutic targets for the development of effective stroke therapy.

DL-3-*n*-Butylphthalide (NBP) is a synthesized compound based on the pure component, L-3-*n*-butylphthalide, originally extracted from the seeds of *Apium graveolens* Linn [12]. NBP, as a neuroprotective drug against ischemic brain, has passed clinical trials as a therapeutic drug for stroke in China [13]. In the randomized double blind trial, a total of 573 patients receiving NBP within 48 hours of the onset of ischemic stroke and 90-day treatment (followed by aspirin) had significantly improved outcomes measured by the modified Rankin scale (mRS) [14]. The control for the comparison was a 14-day infusion of ozagrel followed by aspirin. The chronic treatment of NBP (both intravenous and oral) is judged to be safe and superior to the treatment of sodium ozagrel and aspirin for acute ischemic stroke patients [14]. Chinese Guidelines for the Management of Ischemic Stroke (www.cma.org.cn) recommends NBP capsule for the treatment of acute ischemic stroke [15]. Despite this, it is essential to verify the NBP efficacy against ischemic stroke and long-term functional benefits. Moreover, a better understanding of the mechanism underlying an acute as well as chronic effect of the NBP treatment is critical for basic research and clinical translation of the NBP-mediated therapy.

Greater density of cerebral blood vessels in the ischemic border has been shown to correlate with longer survival of stroke patients [16, 17]. The growth and remodeling of pre-existing parenchymal arterioles (PAs) into physiologically relevant arteries (arteriogenesis) and the growth of new capillaries (angiogenesis) are able to support restored perfusion in the post-ischemia brain and promote long-term recovery. A clinical study shows that the level of circulating CD34/prominin-1(CD133)/VEGFR-2-triple positive vascular endothelial progenitor cells are significantly increased in stroke patients receiving NBP treatment compared with controls [18]. These patients have improved NIHSS score on post-stroke Day 90. One of the known mechanisms seems primarily relaying on the eNOS signaling because eNOS^-/-^ mice show less arteriogenesis and more severe neurological deficits. Treatment with nitric oxide (NO) donor can increase arteriogenesis and improve functional outcome after stroke [19]. Thus, stimulating arteriogenesis may provide a treatment strategy for patients with stroke [20]. By definition, a collateral artery serves as an accessory part or a side branch of a blood vessel [21], opens to shunt blood around the blockage during and after ischemia. Several vascular risk factors may limit this endogenous vessel capacity in the adult brain, especially during aging when there are collateral rarefaction and deficient recruitment [22]s Since NBP was shown to enrich vascular factors bFGF, HIF-1α, and VEGF to increase angiogenesis and reduce neurovascular inflammation after stroke [23-25], we hypothesized that NBP could promote post-stroke arteriogenesis including collateral arteries as a underlying mechanism for its protective and regenerative effects.

In this study, we examined the effects of intranasally deliveried NBP on arteriogenesis of collateral arteries, i.e., collateriogenesis, and functional recovery after stroke. To particularly evaluate the changes in artery formation, a focal cerebral ischemia was induced in the α-smooth muscle actin (αSMA)-GFP transgenic mouse where GFP is expressed specifically under the αSMA promoter to illustrate arterial and arteriolar constructions.

## MATERIALS AND METHODS

### Vascular cell cultures derived from mouse iPSCs

Mouse induced pluripotent stem cell-derived vascular progenitor cell (iPSC-VPC) were differentiated from iPSC originally generated from the embryonic fibroblasts (Stemgent Inc., Cambridge, MA). Use of an inhibitor cocktail of small molecules over two weeks allowed iPSCs to maintain extended pluripotency [26], which preferentially had mesoderm and ectoderm potentials in the dish. iPSCs were cultured in the medium freshly prepared from the media stock (MS) solution that uses the Minimum Essential Medium Eagle (MEM) as basal media with some additional nutrients, modified from our previous publication [27]. The MS solution had adjusted volumes of 9.2% (v/v) 10XMEM (Sigma Aldrich, MO, USA) and 8.3% (v/v) GB stock solution with ddH2O (44.44 g/L glucose and 26.66 g/L NaHCO3, with adjusted pH 7.4). Cells were cultured under 20% O_2_, 5% CO_2_, at 37°C. The modified mouse embryonic stem cell sphere induction medium (mESIM) contained 81% MS, 7% ES qualified FBS, 7% newborn calf serum (NCS; Sigma Aldrich), 1% GlutaMAX (Thermo Fisher Scientific), 1.5% 1M HEPES stock (Sigma Aldrich), and 1% nonessential amino acids (Sigma Aldrich). It was further supplemented with 0.1 mM beta-mercaptoethanol (β-ME; final concentration: 0.1 mM; Sigma Aldrich), nucleoside mix (cytidine 0.73 g/L, guanosine 0.85 g/L, uridine 0.73 g/L, adenosine 0.8 g/L, thymidine 0.24 g/L; Sigma Aldrich) 100 U/mL penicillin/streptomycin (Sigma Aldrich), and sodium pyruvate (1 mM; Sigma Aldrich). Small molecules were recombinant leukemia inhibitory factor (LIF; 10 ng/mL; Millipore, Billerica, MA), CHIR 99021 (3 mM; Tocris), (S)-(þ+)-dimethindene maleate (2 mM; Tocris) and minocycline hydrochloride (2 mM; Santa Cruz Biotechnology, Santa Cruz, CA). For vascular differentiation, iPSCs with LCDM induction for over two weeks were cultured in our “10+10” medium (originally designed for bone marrow mesenchymal stromal cells) containing 79% MS, 10% fetal calf serum (FCS; Sigma Aldrich), 10% horse serum (HS; Sigma Aldrich), 0.25% GlutaMAX (Thermo Fisher Scientific), and epidermal growth factor (EGF, 20 ng/ml; Peprotec, Rocky Hill, NJ).

### Focal cerebral ischemic stroke model in the mouse

A focal cerebral ischemia was induced by permanent occlusion of the distal branch of the right middle cerebral artery (MCA) in adult male mice (2-3-month age with C57BL/6 background, weighing 24-25 g) expressing GFP under the control of the αSMA promoter (αSMA-GFP) [28, 29]. This transgenic mouse was obtained from the Transgenic Mice Facility of the National Eye Institute, NIH (Bethesda, MD). Briefly, animals were subjected to anesthesia with 3% isoflurane and maintained using 1.5% isoflurane during surgery. The distal branch of the right MCA was permanently ligated by a 10-0 suture (Surgical Specialties CO., Reading, PA). The creation of the ischemic insult to the right sensorimotor cortex was completed by bilateral occlusion of the common carotid arteries (CCA) for 7 min. During surgery and recovery periods, body temperature was monitored and maintained at 37.0±0.5°C using a temperature control unit and heating pads. All animal experiments and surgery procedures were approved by the Institutional Animal Care and Use Committee (IACUC) and met NIH standard. The mortality rate of the focal ischemic surgery is less than 5% 3 days after stroke. Most stroke mice can survive for at least up to 28 days after stroke. The infarct formation and functional deficits were relatively stable so rarely any outlier need to be removed from experimental groups.

### Intranasal administration of NBP

Intranasal drug delivery [30] and the dosage justification of NBP were performed as previously described with minor modifications [31]. Five microliter drops of NBP or vegetable oil were carefully placed on one nostril, allowed to be snorted and switching the nostrils after each 1 min. Animals were conscious during the procedure. DL-3-*n*-Butylphthalide (NBP, 80 mg/kg in 400 μl vegetable oil; obtained from Shijiazhuang Pharmaceutical Group Ouyi Pharma Co., Ltd, Shijiazhuang, China) or the same volume of vegetable oil (Kroger, Atlanta, USA) was administrated through the non-invasive intranasal route 1 hr after the stroke onset and once daily until sacrifice.

### 5-bromo-20-deoxyuridine administration

To label proliferating cells, 5-bromo-20-deoxyuridine (BrdU) (Sigma, St Louis, MO) was administrated to all animals (50 mg/kg, intraperitoneal injection) beginning on day 3 after stroke and continued once daily until sacrifice. The experimental time point of 14-days was chosen for studying the regenerative events including arteriogenesis.

### Collateral arterial diameter measurement

Under a fluorescent microscope, brain sections from αSMA-GFP mice show GFP fluorescent signals of artery distributions of αSMA expression. After sacrifice, fresh brains were immediately examined by fluorescent photographing under the FITC (green) excitation wavelength in an Olympus fluorescent microscope (BX61; Olympus, Tokyo, Japan). Photoshop Professional was used to make an image mosaic (Adobes Photoshops CS 8.0, San Jose, CA). The artery diameter was measured using the imaging software Image J (NIH). Six collaterals and six areas of each collateral arteries were measured in each assay. Measurement on MCAO branches was also performed and compared at different levels of arteries. Similar counting and diagram were reported in a previous paper [32].

### Western blot analysis

We previously demonstrated in this ischemic stroke model that vascularature cells and growth factor expressions can remain in the ischemic core for 7-14 days after stroke [33]. The present investigation examined protein expressions in the core and peri-infarct regions at delayed time points. Tissue samples were taken from the ischemic and peri-infarct regions of the cortex and proteins were extracted by homogenization in protein lysis buffer (25 mM Tris-HCl (pH 7.4), 150 mM NaCl, 5 mM EDTA, 0.1% SDS, 2 mM sodium orthovanadate, 100 mM NaF, 1% Triton X-100, leupeptin, aprotinin, and pepstatin). Protein (30 μg) from each sample was loaded into a gradient gel and run at constant current until protein markers had adequately separated. They were transferred onto polyvinyl difluoride membranes that were then probed by using standard protocols [29]. Primary antibodies VEGF (1:100; Santa Cruz, Dallas, TX), Ang-1 (1:500, Abcam, Cambridge, MA), nNOS (1:400; EMD Millipore, Burlington, MA), Tie-2 (1:500; Santa Cruz) and mouse β-actin antibody (1:6000; Sigma, St. Louis, MO) were applied overnight at 4°C. Alkaline phosphatase (AP)-conjugated secondary antibodies were applied for 1 to 2 hrs at room temperature. AP-developed bands were developed on the membrane bathed in nitro-blue tetrazolium and 5-bromo-4-chloro-3’-indolyphosphate (NBT/BCIP) solution. The intensity of each band was measured and subtracted by the background using the NIH Image J software. The expression ratio of each target protein was then normalized against β-actin. We used this ratio to compare experimental groups with the sham group to describe the changes of protein levels after ischemic stroke and NBP treatment.

### Immunocytochemical and immunohistochemical staining in cultured cells and brain sections

In the *in vitro* experiment with cultured VPCs divergent from mouse iPSCs, CD-31 (1:1,000; Fitzgerald), PDGFRα, αSMA and p-VEGFR2 (1:1,000, Novus Biologicals) were stained for differentiated αSMA-positive vasculogenic cells. The area of positive staining was measured using the fraction area measurement function of the Image J software (NIH).

In immunohistochemical experiments, formalin (10 %) fixed brain slides with a thickness of 10 μm were stained for NeuN (1:400; Millipore, Billerica, MA) to label neurons, GFP (1:200; Novus Biologicals, Littleton, CO) for αSMA-positive arteries, or BrdU (1:400; AbD Serotec, Oxford, UK) to label newborn cells. Based on the NeuN staining, the peri-infarct region was morphologically determined in the regions adjacent to the stroke core. Staining was visualized by a fluorescent microscopy (Olympus, Japan). Z-stack imaging was used to confirm co-localization using the Olympus Stream. For systematic random sampling in design-based stereological cell counting, six coronal brain sections per mouse were selected, spaced 90 µm apart across the region of interest in each animal. For multistage random sampling, six fields per brain section were randomly chosen in the peri-infarct/penumbra regions of the brain. All counting assays were performed under blind condition.

### Local Cerebral Blood Flow (LCBF) measurement

Laser scanning imaging was used to measure LCBF as previously described [34]. LCBF was measured at different time points: immediately before MCA ligation, right after MCA/CCA occlusion, after the 7 min bilateral CCA ligation, and 14 days after ischemia. Animals were anesthetized with an injection of 3% isoflurane and an incision was made to expose the skull above the territory of the right MCA. The laser was placed over the center of right coronal suture. Different from the conventional Laser Doppler probe that measures a small point of blood flow, the scanner method measures a 2.4×2.4 mm square area using the Laser Doppler perfusion imaging system (PeriFlux System 5000-PF5010 LDPM unit, Perimed, Stockholm, Sweden). This scanning measurement largely avoids inaccurate or bias results caused by inconsistent location of the laser probe. Data was analyzed using the LDPI Win 2 software (Perimed AB).

### Behavior tests

The corner test and cylinder test were performed according to our previous methods [35]. In the corner test, two cardboard plates (30 cm × 20 cm × 0.3 cm) were attached at a 30° angle from each other in a home cage. Each subject mouse was placed between the two plates and allowed to freely move to the corner within the first five minutes. The number of right and left turns was counted. In the cylinder test, the mice were placed in a glass cylinder (9.5 cm diameter and 11 cm height), and the number of times each forelimb or both forelimbs were used to support the body on the wall of the cylinder was counted within the first five minutes. The number of impaired and nonimpaired forelimb contacts was calculated as a percentage of total contacts.

The adhesive removal test measured sensorimotor function as previously described [36]. A small adhesive dot was placed on each forepaw, and the time (seconds) needed to contact and remove the sticker from each forepaw was recorded. Recording stopped if the animal failed to contact the sticker within 2 min. Mice were trained three times before stroke surgery to ensure that they were able to remove the tape. The test was performed three times per mouse, and the average time was used in the analysis at before stroke and 14 days after stroke.

The Von Frey nocioperception assay measured sensitivity to a mechanical stimulus induced by von Frey nylon rod of varying diameters in the mice. The animal stood on an elevated mesh platform, and the Von Frey nylon rods were inserted through the mesh to poke their forepaws.

Reciprocal social interaction test measured the interactions between test mice and unfamiliar, weight-matched, age-matched, and sex-matched stranger mice partners [37]. The social behavior measure was used as one of the cognitive and psychological functional recovery in the rodent, previously shown in our study [38]. Mice were tested in a clean empty home cage. Time spent in social interaction between the animals was recorded within the ten minutes after a prior 10-min habituation to odors in the cage.

### Statistical analysis

The Student t test was applied to compare two groups. Multiple comparisons were done using one-way ANOVA followed by Tukey test or two-way ANOVA followed by Bonferroni test. Significances were identified if *P* value was less than 0.05. Mean values were reported together with the standard error of the mean (SEM).

**Figure 1.**
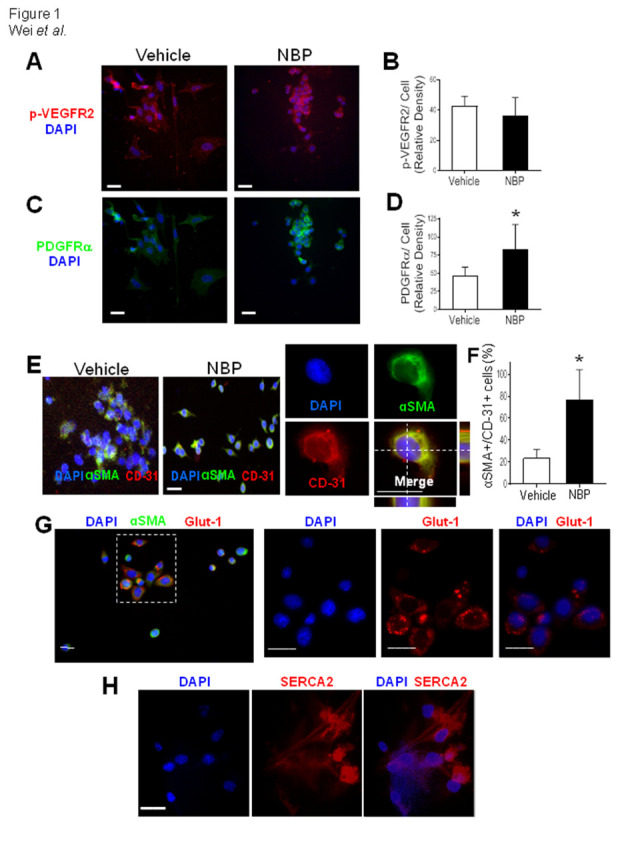
NBP increased endothelial cell differentiation and αSMA expression in vitro. Immunocytochemical images of cultured mouse iPSC-derived vascular progenitor cells positive to CD-31, PDGFRα, αSMA, and p-VEGFR2 expression 48 hrs after 10 μM NBP or vehicle treatment. (A) - (D) The relative density of immunofluorescence of p-VEGFR2 was similar (A) and (B), while the expression of PDGFR2 was significantly increased in NBP-treated cells (C) and (D). (E) and (F) The NBP treatment increased differentiation of αSMA and CD-31 double positive cells in endothelial progenitor cells. 3-D images demonstrate the co-localization of these two markers. (G) and (H) These cells were also positive to immunostaining of the vascular marker Glut-1 (G) and the calcium regulatory pump SERCA2 (H). *p<0.05 vs. vehicle controls. N=3 independent cell batches per group. Bar=20 μm.

## RESULTS

### NBP promoted vacular endothelial differentiation in iPSC cultures

In cultured vascular endothelial progenitor cells derived from mouse iPSCs, immunocytochemical staining revealed that NBP (10 μM) exposure for 48 hrs significantly increased the expression of newly formed vascular marker PDGFRα [39], while the phosphorylated VEGFR2 (p-VEGFR2) remained the same (Fig. 1A - 1D). Interestingly, expressions of αSMA and CD-31, the vascular smooth muscle and endothelial markers, respectively, co-existed in many cells, indicative of endothelial progenitor cells [40]. Exposure to NBP for 48 hrs significantly enhanced the percentage of αSMA/CD-31 double positive cells from 20-25% to more than 70% of total cells (Fig. 1E and 1F). Consistently, around 80% of αSMA-positive cells also expressed Glut-1, which is another vascular endothelial marker (Fig. 1G). Moreover, sarco/endoplasmic reticulum Ca^2+^-ATPase (SERCA2) was observed in all of these cultured cells, suggesting the regulatory capability of Ca^2+^ homeostasis of these differentiating vascular cells (Fig. 1H) [41].

**Figure 2.**
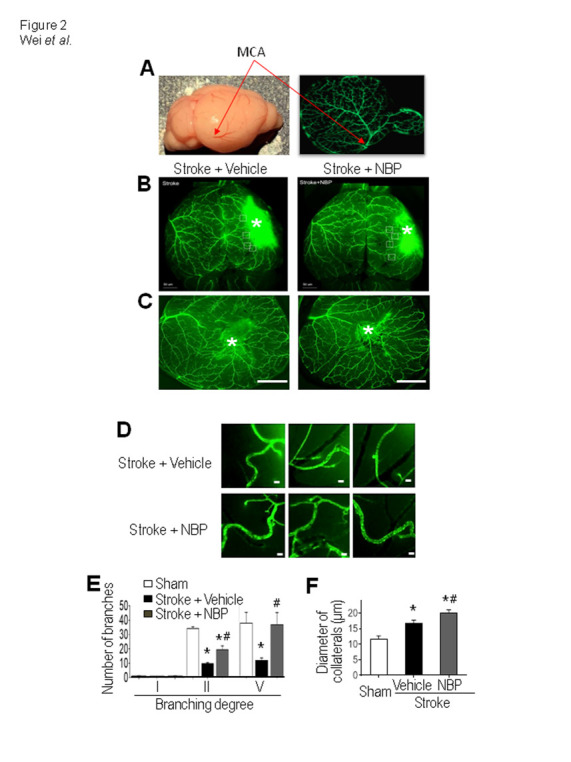
NBP increased collateral growth in the ischemic cortex. (A) The whole brain photo and the GFP green fluorescence image show the middle cerebral artery (MCA) and the surface vasculature distributions in the mouse brain, used to measure MCAO branches at 3 days after stroke. (B) and (C) Representative images showing main and collateral arteries in the ipsilateral hemisphere. Images show the surface of the cortical area and the ischemic core region (*). Bar=1 mm. (D) Enlarged images of collateral arteries in the peri-infarct region. The diameter of collaterals was measured 14 days after stroke. Bar= 20 μm. (E) and (F) Quantified summary of the image analysis of the number and diameter of collaterals. The vessels over the surface were examined and compared. The ischemic insult caused an increase in the number and diameter of the collateral artery. Stroke animals received NBP treatment exhibited even greater increases in the arterial number and diameter. One-way ANOVA; F(2,21)=0.4134 for branching degree I, F(2,21)=38.62 for II, F(2,21)=3.988 for IV, and F(2,10)=21.27 for the diameter, *p<0.05 vs. sham, *^#^ p<*0.05 vs. stroke + vehicle. N=3 in sham group, N=5 in stroke + vehicle group and stroke stroke + NBP group.

### Focal ischemia-induced cortical damage and NBP enhanced collateriogenesis after stroke

**Figure 3.**
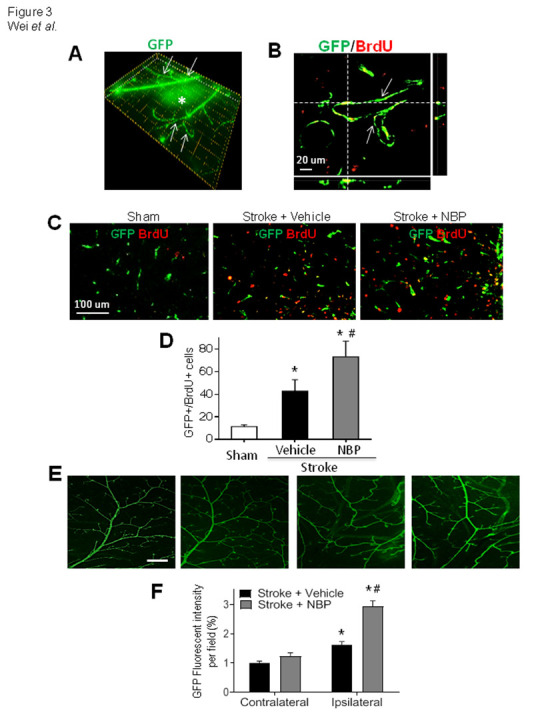
NBP enhances arterial proliferation after ischemic stroke in vivo. Imaging examinations of *α*SMA-GFP and proliferation marker BrdU in the brain of 14 days after stroke. (A) Collateral arteries (arrows) and the ischemic region (*) can be visualized over the surface of the sensorimotor cortex. Each frame (dotted lines)=20X20 μm. (B) The confocal 3-D image shows vascular smooth muscle cells transgenically labeled with *α*SMA-GFP (green) and stained for the proliferation marker BrdU (red) in the peri-infarct region. Those vascular cells of collateral arteries co-labeled with GFP and BrdU (yellow) were counted as newly formed vasculature. (C) and (D) Representative images from sham and stroke animals and quantified data of GFP/BrdU co-labeled cells in stroke animals with and without NBP treatment. *p<0.01 vs. sham, #p<0.05 vs. stroke + vehicle, N=6 in sham group, N=8 in stroke + vehicle and stroke + NBP groups, respectively. One-way ANOVA, F(2,14)=33.82. Bar=20 μm. (E) and (F) Representative images and the percentage of GFP expression per field (area fraction) was quantified by image analysis. The ischemic insult caused increased arteriogenesis in the peri-infarct region of vehicle or NBP group compared with sham or contralateral side. The NBP treatment promoted the expression of PDGFRα, a marker for newly formed vasculatures, compared to vehicle controls. Bar= 500 μm. N=6 in sham group, N=8 in stroke + vehicle group and stroke + NBP group, respectively. Two-way ANOVA followed by Bonferroni test; *p<0.05 vs. contralateral, #p<0.05 vs. stroke + vehicle.

The NBP effect on arteriogenesis was then tested *in vivo* using a focal ischemic model of selective damage to the sensorimotor cortex of the αSMA-GFP transgenic mouse [28, 29]. Fourteen days after stroke, brain sections were subjected to immunohistochemical staining for identifying pre-existing and newly formed arteries. In the dissected intact brain of the mouse, large and smaller arteries/arterioles could be visualized on the surface of the cortex (Fig. 1A). Collateral artery is an accessory part or a side branch of a blood vessel [42], which can open to shunt blood around the blockage during and after ischemia. We took the advantage of the αSMA-GFP marker in the transgenic mouse to identify collaterals according to the distribution pattern of GFP fluorescence. The GFP-positive arterioles on the surface of the brain were imaged and then the images were made to a mosaic (Fig. 2B and 2C). Specifically, MCAO branches were measured according to different levels of branching at 3 days after stroke (Fig. 3D and 3E). The diameter of all collaterals around the infarct was analyzed in each group at 14 days after stroke (Fig. 2F). The number of MCAO branches measured at branching degree II and V after NBP treatment was significantly increased in the stroke animals at 3 days after stroke (Fig. 3E). At 14 days after stroke, the diameter was 11.8±0.89 μm and 16.9±0.82 μm in sham and stroke vehicle groups, respectively. The NBP treatment further increased the diameter of collaterals in the ipsilateral cortex to 20.23±0.81 μm (*P*<0.05 vs. stroke vehicle group) (Fig. 2F).

Newly generated smooth muscle cells in ischemic and peri-infarct regions, indicated by αSMA-GFP and BrdU co-labeling, were counted to determine arteriogenesis after stroke (Fig. 3A - 3C). Significantly more GFP/BrdU-double labeled cells were identified in stroke mice received NBP treatment (80 mg/kg, intranasal delivery, 1 hr after stroke and once daily thereafter; Fig. 3C and 3D). The NBP treatment significantly increased the artery density in the ischemic and peri-infarct regions; no difference was seen in the contralateral cortex between control and NBP groups (Fig. 3E and 3F).

**Figure 4.**
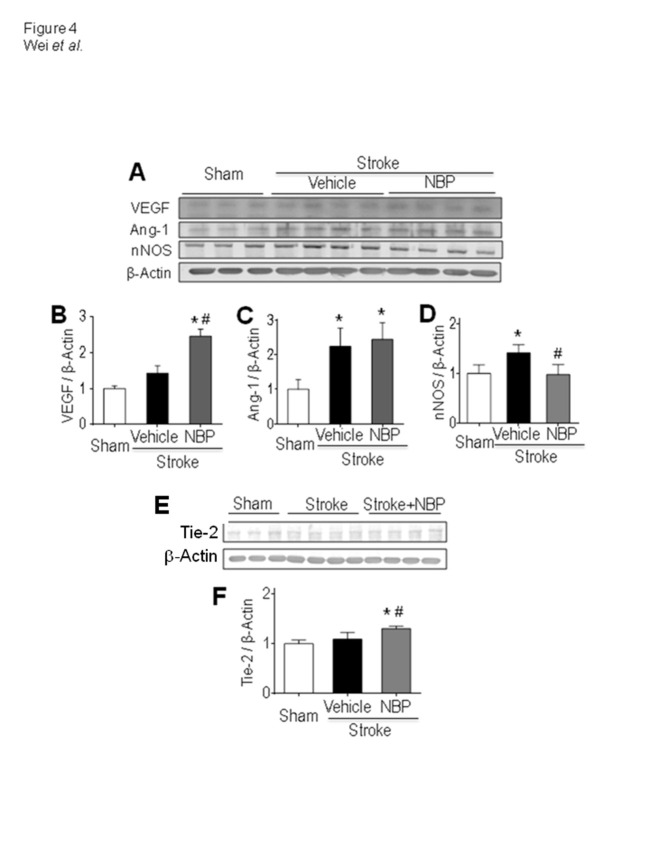
Effects of NBP on neurovascular factors in the ischemic cortex. (A) The protein level of neurovascular regulatory factors VEGF, Ang-1, Tie-2 and nNOS in the peri-infarct region were detected by Western blot analysis 14 days after stroke. (B) Quantified data showed that, at this delayed time point, the VEGF level in the post-stroke brain was similar to that in sham controls while the NBP treatment significantly increased the VEGF expression. One-way ANOVA; F(2,8)=16.26, *p<0.05 vs. sham control, #p<0.05 vs. stroke+vehicle control, N=6 in sham group, N=8 in stroke and stroke plus NBP, respectively. (C) Stroke significantly enhanced the expression of Ang-1; there was no significant difference between stroke and stroke plus NBP group. One-way ANOVA; F(2,19)=19.07, *p<0.05 vs. sham, N=6 in sham group, N=8 in stroke vehicle and stroke plus NBP group, respectively. (D) The expression of nNOS in stroke vehicle animals increased compared to sham controls. Stroke animals received the NBP treatment, however, did not show similar increase. (E) and (F) The Tie-2 level was significantly increased by NBP. One-way ANOVA; F (2, 11) = 11.49; *p<0.05 vs. sham, #p<0.05 vs. stroke vehicle controls. N=6 in sham group, N=8 in stroke + vehicle group and stroke stroke + NBP group, respectively.

### NBP increased VEGF expression after stroke

To elucidate the possible mechanism of the pro-arteriogenesis effect of NBP, we evaluated several vascular regulatory factors in the ischemic and peri-infarct regions. The protein expression of vascular endothelial growth factor (VEGF), angiopoietin-1 (Ang-1), and nNOS were inspected 14 days after stroke (Fig. 4A). The Western blot analysis showed that, at this delayed time point, the VEGF level in stroke mice was not significantly different from sham controls (Fig. 4A and 4B). The 14-day NBP treatment, however, significantly increased VEGF in the peri-infarct region, implying a persistent support of vascular regenerations (Fig. 4A and 4B). The expression of Ang-1 similarly increased in the stroke control and stroke plus NBP groups (Fig. 4A and 4C). The stroke insult enhanced nNOS levels, consistent with the known neurotoxic role of nNOS [43, 44], while the NBP treatment significantly decreased the nNOS expression compared with stroke controls (Fig. 4A and 4D). The Tie-2 level was significantly higher in NBP-treated mice compared to stroke controls (Fig. 4E and 4F).

### Effect of NBP on LCBF after stroke

**Figure 5.**
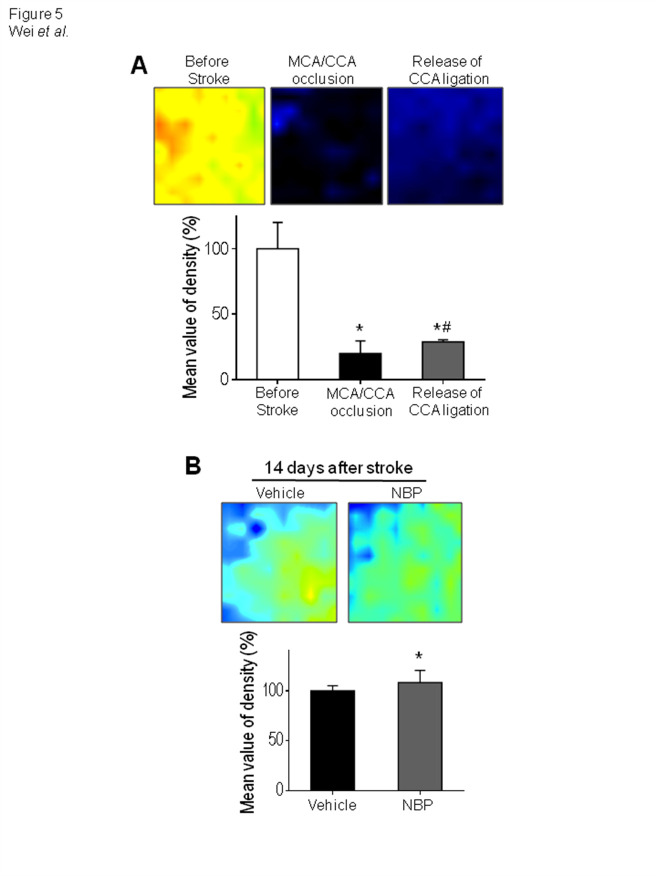
NBP enhanced LCBF restoration after ischemic stroke. (A) The local cerebral blood flow (LCBF) in the ischemic and peri-infarct regions was measured before, during and 14 days after stroke using a laser Doppler image scanner. The restoration of LCBF in each group was calculated by the percentage of the mean value of LCBF to the value before stroke. Stroke animals showed reduced flow in this region during ischemic surgery. (B) LCBF in the peri-infarct and core regions showed a spontaneous recovery 14 days after stroke, the NBP treatment promoted the LCBF restoration. One-way ANOVA; F(2,14)=4.655, *p<0.05 vs. sham. N=6 in sham group, N=8 in stroke + vehicle group and stroke stroke + NBP group.

Since local blood flow is essential to deliver oxygen and nutrition for sustained cell survival and tissue repair after stroke, LCBF was measured using Laser Doppler imaging (Fig. 5A). Fourteen days after stroke, stroke control animals showed about an 80% LCBF recovery in the ischemic and peri-infarct regions (Fig. 5B). A 90% restoration of LCBF was seen in similar regions of stroke mice received NBP treatment. LCBF in NBP-treated mice showed no difference from sham animals (data not shown).

**Figure 6.**
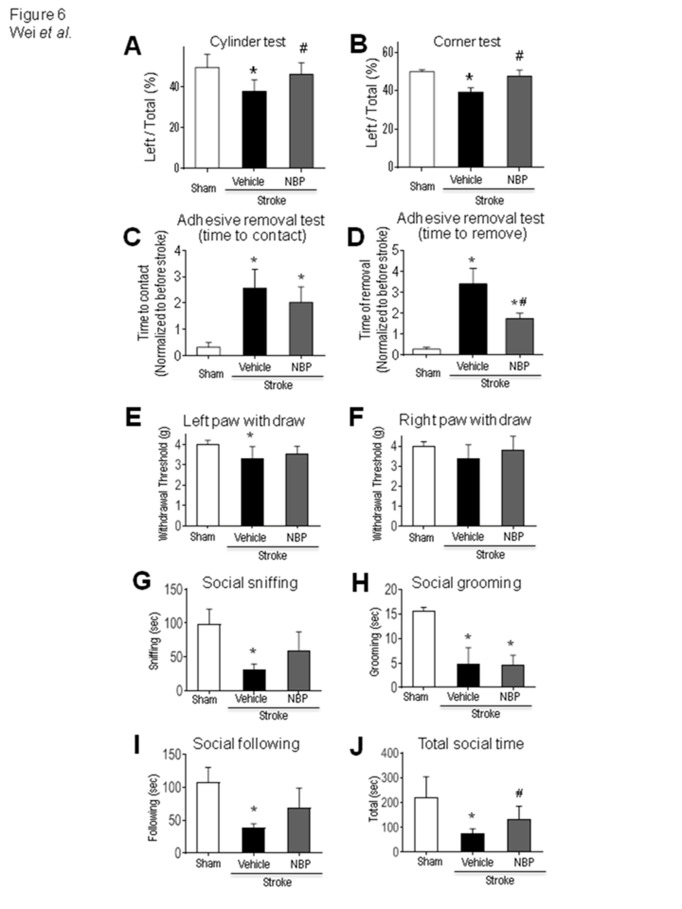
NBP attenuated acute and chronic functional and behavioral deficits after stroke. Functional tests were performed at 3 and 14 days after stroke to evaluate acute and long-term effects of NBP treatments. (A) and (B) Three days after the ischemic insult, cylinder test (A) and corner test (B) revealed an early functional benefit, consistent with the acute protective effect of NBP. One-way ANOVA followed by Bonferroni test; *p<0.05 vs. sham, ^#^ p<0.05 vs. stroke + vehicle, N=8 per group. Data are shown as mean±SEM. Fourteen days after stroke, sensorimotor function and social behaviors were tested for long-term effects of repeated NBP daily treatments. (C) The adhesive removal test assessed sensorimotor function. The time needed for stroke animals to feel (time to contact) the sticky dot from the left paw corresponding to the right cortical damage was significantly increased compared to sham animals. One-way ANOVA, F(2,16)=18.35, *p<0.05 vs. sham. (D) The time for the animal to remove the dot (time to remove) was also significantly prolonged. The NBP treatment significantly shortened the time in this test. One-way ANOVA, *p<0.05 vs. sham, #p<0.05 vs. stroke vehicle, N=6 in sham group, N=8 in stroke + vehicle group and stroke + NBP group, respectively. (E) and (F) Von Frey forepaw withdraw test. Stroke mice show decreased withdrawal threshold in the forepaw corresponding to the damaged sensorimotor cortex while mice received NBP did not have this reduction. *p<0.05 vs. sham. N=8 per group. (G) - (J). Time spent in social sniffing, social grooming, social following, and total social time were significantly reduced in the stroke mice. The stroke insult caused great reductions in all these social activities, while the social sniffing and social following were not significantly deteriorated in the NBP group compared to sham controls. The NBP treatment significantly improved the total social time of all social activities compared to stroke vehicle controls (J). *p<0.05 vs. sham, #p<0.05 vs. stroke vehicle. N=10-12 per group.

### NBP improved acute and delayed functional and behavioral performance after stroke

Our previous data demonstrated significantly enhanced neuronal survival after NBP administration [45]. Consistently, the NBP treatment showed behavior improvements starting at 3 days after stroke. At the early time point, stroke animals had reduced left paw usage measured in the cylinder test and reduced whisker sensory-related left turns measured in the corner test. The NBP treatment increased the left limb use and correct for the turn bias (Fig. 6A and 6B).

Fourteen days after stroke, several tests were performed to assess delayed functional and behavior deficits. The adhesive removal test was used to assess sensorimotor function. The time needed for stroke animals to feel (time to contact) and remove the sticky dot from the left paw (time to remove) was markedly prolonged after stroke due to the central damage in the corresponding sensorimotor pathway (Fig. 6C and 6D). Stroke animals took 3-4 times longer to contact and remove the sticky dot from their left paw compared to their own performance before stroke or sham controls. This time for removing the sticky dot was significantly shortened by the NBP treatment (Fig. 6C and 6D). The Von Frey test revealed that stroke mice showed enhanced sensation to mechanical stimuli, the NBP treatment helped to attenuate this defect (Fig. 6E and 6F). In addition, we also detected beneficial effects of the NBP treatment on social cognition deficits developed after stroke. In social interaction tests, stroke mice had significantly lower social sniffing, grooming, following behaviors, resulting in shortened total social time spent with test strangers (Fig. 6G-6J). The NBP treatment significantly increased the total social time after stroke (Fig. 6J).

## DISCUSSION

The present investigation demonstrates that NBP treatment after ischemic stroke increases the expression of several regenerative factors and arteriogenesis in ischemic and peri-infarct regions, helping to restore LCBF and functional activity in stroke mice. Utilizing the αSMA-GFP transgenic mouse, this study provides new evidence of the collateral growth after stroke, which is expected to contribute to the LCBF restoration after stroke. Our data suggest that NBP shows beneficial for acute and long-term protection partially through arteriogenic effects.

In a rat model of MCA occlusion, NBP administrated at 1 and 4?hrs prevented the vasoconstriction of the artery measured by synchrotron radiation angiography and maintained its diameter at normal level [46]. A randomized controlled STROBE study has tested the efficacy and safety of Dl-NBP on progressive cerebral infarction cases. NBP showed signs of promoting establishment of collateral circulation and enhancing cerebral blood flow [47]. In our investigations, we focused on "collateriogenesis" after stroke as several clinical trials of ACUTE thrombectomy identified that collateral circulation around the ischemic core region could be an important factor in determining outcomes. The current report did not focus on an acute phase (no time points earlier than Day 3) or neuronal cell death. Instead, this study provides the important data for later time points (such as Day 14) to support restoration of LCBF and sustainable positive outcomes. NBP was long recognized as a neuroprotective drug in acute and chronic conditions [48, 49]. The mechanistic reason for focusing on the possible association of NBP and collateral circulation was based on the roles of NBP in robust regulation of several vascular regenerative factors [50]. The focuse on long-term effects including flow recovery was independent of the lesion size or acute neuroprotection. Increased collateral circulation, however, is expected to show synergic effects with enhanced cell survival and long-term benefits on psychological deficits that may chronically develops after stroke [51].

Arteriogenesis refers to the formation of new arterioles and these newly formed or preexisting arterioles transform into constructions of larger diameters [52, 53]. The collaterals act as a first vasculature reaction against tissue ischemia, by providing alternative pathways for arterial blood supply. The collateral circulation compensates the oxygen and other nutrients to ameliorate cerebral ischemic damage in the brain [54-56]. Basic and clinical studies show that sufficient collateral circulation is critical for neuronal and tissue survival and repair [57-59]. However, spontaneous growth of collateral artery is slow, self-limiting and not sufficient for stroke recovery. Thus, stimulating collateriogenesis is a potential therapeutic target for stroke treatment [60]. Many factors thought to promote arteriogenesis have been reported, which include bFGF, GM-CSF, monocyte chemoattractant protein 1, PDGF-BB, placental growth factor, and TGF-β1. Delivery of exogenous angiogenic growth factors such as VEGF and FGF has been proposed as a therapeutic strategy to augment the artergiogenesis after ischemia [61-63]. Stroke animals received NBP treatment showed even larger collaterals and more proliferating αSMA-positive smooth muscle cells. Western blotting data showed that NBP significantly increased the expression of VEGF and Ang-1. The observation is consistent with other reports that NBP increased bFGF, VEGF/VEGFR-2 and improved vessel growth in ischemic rats [64]. The enhanced signaling may further promote the vascular endothelial cell differentiation. Our data support that the NBP treatment significantly increased newly generated endothelial cells labeling with BrdU and endothelial cell-related markers including Glut-1, ColIV, and CD-31 [65].

Arteriogenesis can be regulated by other angiogenic factors such as nitric oxide (NO) and shear stress [19, 66, 67]. NO production (e.g. by endothelial cell-related eNOS) is also critical to the efficacy of therapeutic arteriogenesis achieved by delivery of exogenous angiogenic growth factors. NBP has been shown to exhibit the vasodilatory effects through increasing the vascular NO production, and to increase the number of cerebral microvessels via up-regulated VEGF and hypoxia induced factor-1α (HIF-1α) [24]. We showed before that intranasal NBP treatment increased neurogenesis, angiogenesis, and arteriogenesis in the post-TBI brain, accompanied with upregulations of BDNF, matrix metallopeptidase 9 (MMP-9), eNOS, and VEGF [68]. In the current study, we additionally examined the effect of NBP on the expression of nNOS that is believed to be neurotoxic in ischemic injuries [43, 44]. An ischemic insult increased the nNOS level, likely played an injurious role in an ischemic brain. The nNOS level was reduced by NBP treatment, which is consistent with the protective action of NBP.

The *in vitro* and *ex vivo* analysis on the morphological change of vasculatures suggested that the significant increase in arterial diameter was likely due to proliferative VSMC instead of the wall shear stress. In the study on the number of small arteries in the ischemic core and penumbra regions that would be collateral circulation supply, we were able to define the collateral artery by using the αSMA-GFP transgenic mouse. This was an important step visualizing small arteries and arterioles in 2-D and 3-D imaging. A total of small arteries and arteriole were measured and, specifically, the number of MCA branches distinguished from the normal vessel was counted based on collateral recruitment features and information in our recent investigation [69].

In the present investigation, NBP was administered via the intranasal delivery route. The intranasal method for delivering experimental and therapeutic neuropeptides, drugs and even stem cells to the brain has been verified by many studies from our and other groups [30, 31, 38]. Intranasally delivered substance can cross the cribriform plate connecting to the nasal cavity to the olfactory bulb of the forebrain and migrate/diffuse to injuried brain regions as early as 2 hrs after the administration [70, 71]. This non-invasive drug administration should significantly facilitate clinical feasibility of repeated daily drug therapy for stroke patients.

Different from many traditional Chinese herbal medications, the tested NBP is synthesized based on the pure component extracted from seeds of celery *Apium graveolens Linn*. Its efficacy and safety have been proved in a randomized, double-blind, double-dummy clinical trial (ChiCTR-TRC-09000483) [14]. The present study provides an additional evidence for a beneficial arteriogenic effect of NBP, supporting that NBP may be considered in stroke treatments for short-term protection and long-term functional and behavioral recovery.
